# Early-Onset Colorectal Cancer: From Epidemiologic Shift to Life-Course Carcinogenesis and Age-Attuned Care

**DOI:** 10.1007/s11912-026-01815-1

**Published:** 2026-08-20

**Authors:** Magda Grześkiewicz-Szostak, Katarzyna Gęca, Magdalena Skórzewska

**Affiliations:** Department of Clinical Oncology and Chemotherapy, Medical University of Lublin, Lublin, Poland

**Keywords:** Eearly-onset colorectal cancer, Young-onset colorectal cancer, Ccolorectal cancer screening, Life-course carcinogenesis, Microbiome, Colibactin, Diagnostic delay, Hereditary predisposition, Precision prevention, Survivorship

## Abstract

**Purpose of Review:**

Early-onset colorectal cancer (EOCRC), conventionally defined as colorectal cancer diagnosed before the age of 50 years, has become one of the most important and unsettling epidemiologic shifts in gastrointestinal oncology. This review critically synthesizes recent evidence on EOCRC epidemiology, risk architecture, life-course carcinogenesis, molecular and microbiome-associated mechanisms, diagnostic delay, screening limitations, treatment considerations, and survivorship needs, with the aim of reframing EOCRC as an age-attuned clinical and biological challenge rather than a simple early presentation of conventional colorectal cancer.

**Recent Findings:**

Recent population-based analyses confirm that EOCRC incidence is increasing across multiple countries and birth cohorts, with a disproportionate contribution of distal colon and rectal cancers. These data suggest that the rise of EOCRC is unlikely to be explained by improved detection alone and instead points toward changing generational exposures. Contemporary studies have moved the field beyond hereditary predisposition as the dominant explanatory model: although germline syndromes remain essential to identify, most EOCRC is sporadic or incompletely explained by known inherited risk. Recent literature increasingly implicates metabolic dysfunction, obesity, westernized dietary patterns, early-life exposures, inflammation, antibiotic-associated microbial disruption, and host–microbiome disequilibrium. Particularly important are emerging genomic data linking colibactin-associated mutational signatures to younger-onset disease, supporting the hypothesis that microbial genotoxicity may imprint early driver events long before clinical diagnosis. In parallel, recent clinical studies show that EOCRC is frequently symptomatic, yet diagnosis is commonly delayed because alarm features such as rectal bleeding, abdominal pain, altered bowel habits, and anaemia are often underestimated in younger adults.

**Summary:**

EOCRC is best understood as a heterogeneous, life-course disease shaped by the convergence of inherited susceptibility, environmental and metabolic exposures, microbiome-mediated biology, tumour site, diagnostic-system factors, and survivorship context. Lowering the average-risk screening age to 45 years is necessary but insufficient, because many cases still occur below routine screening thresholds. A modern EOCRC strategy must therefore combine risk-adapted prevention, improved family-history capture, timely investigation of red-flag symptoms, systematic germline and tumour profiling, and treatment planning that accounts for decades of survivorship. Future progress will depend on moving beyond age alone toward integrated models that connect epidemiology, exposome biology, microbial mutagenesis, precision early detection, and age-specific care.

## Introduction

Colorectal cancer (CRC) remains one of the most common malignancies worldwide and has traditionally been regarded as a disease of older adults. However, this paradigm has been increasingly challenged by the rising incidence of early-onset colorectal cancer (EOCRC), usually defined as CRC diagnosed before the age of 50 years [1, 2]. Although overall CRC incidence has declined or stabilized in older populations in several high-income countries, largely owing to screening uptake and improved awareness, incidence in younger adults has continued to rise, making EOCRC one of the most concerning shifts in contemporary gastrointestinal oncology [1, 2].

The conventional definition of EOCRC as disease diagnosed before the age of 50 years remains clinically practical, but it is biologically imperfect. This threshold was historically aligned with screening practices rather than with a validated molecular boundary. Its limitations have become even more apparent since the US Preventive Services Task Force lowered the recommended starting age for average-risk colorectal cancer screening from 50 to 45 years [3]. Although this policy shift represents an important public health response, it does not fully solve the EOCRC problem, because a substantial proportion of cases still arise before the age of 45 years and therefore remain outside the scope of routine age-based screening [3, 4].

Importantly, EOCRC should not be conceptualized simply as “conventional CRC occurring earlier.” Increasing evidence suggests that it represents a heterogeneous group of tumours arising through a complex interplay of hereditary susceptibility, familial aggregation, metabolic dysfunction, dietary and lifestyle exposures, inflammatory conditions, microbiome perturbation, and possibly early-life environmental influences [4–7]. While inherited cancer syndromes such as Lynch syndrome and familial adenomatous polyposis account for an important subset of cases, the majority of EOCRC appears to be sporadic or only partially explained by currently recognized hereditary factors [5, 7]. This observation has shifted scientific attention toward broader life-course models of carcinogenesis, in which host susceptibility interacts with cumulative environmental and microbial exposures over time [4–7].

Among the most compelling recent advances is the growing evidence linking EOCRC to early biological consequences of microbiome-derived mutagenic exposure. A landmark genomic study demonstrated that mutational signatures associated with colibactin-producing bacteria were enriched in EOCRC, being 3.3-fold more common in tumours diagnosed before the age of 40 years than in those diagnosed after 70 years, and were likely imprinted early during tumour development [6]. Colibactin-associated mutagenesis was also linked to early APC driver alterations, supporting the hypothesis that at least a subset of EOCRC may originate through biologic pathways initiated much earlier in life than previously assumed [6]. In parallel, recent reviews increasingly frame EOCRC as an emerging disease of metabolic dysregulation, with obesity, insulin resistance, and type 2 diabetes mellitus being considered plausible contributors to the rising burden observed in younger populations [5].

The clinical implications of these epidemiologic and biologic shifts are substantial. Unlike older adults, younger patients are typically not included in routine screening programs and often enter the diagnostic pathway only after symptoms develop. Yet even when alarm symptoms such as rectal bleeding, altered bowel habits, abdominal pain, anaemia, or weight loss are present, diagnosis may still be delayed because both patients and clinicians frequently attribute these findings to benign conditions [4]. Recent work has emphasized that EOCRC is shaped not only by tumour biology, but also by repeated missed opportunities for earlier detection across the patient journey, including delayed symptom appraisal, reduced clinical suspicion, and barriers to timely referral and colonoscopic evaluation [4].

Taken together, these observations challenge traditional age-centered assumptions in colorectal cancer prevention and diagnosis. The rise of EOCRC suggests that current models remain too heavily calibrated to the epidemiology of older adults, whereas contemporary data increasingly support the need for more nuanced, risk-adapted approaches to prevention, early detection, and management [2–5]. In this context, the present review aims to provide an updated and integrative overview of EOCRC, with particular emphasis on epidemiologic trends, risk architecture, molecular and microbiome-related mechanisms, clinical presentation, diagnostic delay, and implications for early detection and prevention. Rather than treating EOCRC as a simple age-defined variant of CRC, we discuss it as a heterogeneous and increasingly consequential clinical entity that challenges existing paradigms of carcinogenesis, screening, and oncologic care.

## Epidemiology and Global Burden

The most recent epidemiologic analyses indicate that the rise in EOCRC is neither isolated nor anecdotal. A large population-based study including data from 50 countries and territories showed that incidence rates of colorectal cancer in younger adults are increasing in 27 countries, with many regions demonstrating either an exclusive rise in younger age groups or a steeper increase than that observed in older adults [2]. These data suggest that EOCRC represents a global and expanding phenomenon rather than a pattern restricted to a few highly screened Western populations [2]. At the same time, substantial geographic heterogeneity remains, indicating that local environmental, lifestyle, healthcare, and demographic factors probably modulate the magnitude of risk [2].

Recent data from the United States further underscore the scale of this trend. According to the American Cancer Society, colorectal cancer is now the leading cause of cancer death in men younger than 50 years and the second leading cause in women younger than 50 years [1]. In addition, incidence in individuals aged 20–49 years increased by approximately 3% annually between 2013 and 2022, while overall incidence declined by 0.9% per year during the same period, driven largely by falling rates in adults aged 65 years and older [1]. This divergence strongly suggests that EOCRC is not merely a reflection of overall CRC burden, but a distinct epidemiologic problem with its own trajectory [1].

An important feature of this trend is the apparent predominance of distal tumours, especially cancers of the rectum and sigmoid colon. Contemporary American Cancer Society analyses indicate that the recent increase in younger adults has been driven mainly by distal colon and rectal cancers [1, 9]. This anatomic distribution is clinically relevant because distal disease may differ from proximal disease in symptom profile, diagnostic pathway, biology, and therapeutic consequences, especially in younger patients for whom rectal cancer carries substantial long-term functional and quality-of-life implications [9].

One of the most compelling epidemiologic concepts in EOCRC research is the birth-cohort effect. Multiple studies have shown that the rise in EOCRC is more consistent with changing exposures across successive generations than with a simple period effect related to better diagnostics alone [8, 9]. Individuals born in more recent decades appear to carry substantially higher colorectal cancer risk at younger ages than those born earlier, even after accounting for broader temporal changes in healthcare systems [9]. In one recent analysis, the odds of developing EOCRC were reported to be approximately three times higher in adults born in the mid-1980s than in those born in the mid-1960s [10]. This observation is particularly important because it supports the hypothesis that the modern rise in EOCRC is linked to population-level shifts in early-life and long-term exposures, including obesity, ultra-processed diets, sedentary behaviour, antibiotic-associated microbiome perturbation, and other features of westernisation [8–10].

Despite the robustness of these trends, EOCRC still accounts for a minority of all colorectal cancer cases in absolute terms, and its incidence remains substantially lower than that observed in older adults [9]. This point should be stated clearly to avoid overstating the epidemiologic burden. However, the public health significance of EOCRC lies not only in its current absolute numbers, but also in the consistency of its upward trajectory, the younger age at diagnosis, the frequent occurrence outside routine screening pathways, and the large number of life-years lost when disease develops in early or mid-adulthood [1, 8, 9].

Another important epidemiologic observation is that younger adults are often diagnosed at more advanced stages. Although this is partly discussed later in the manuscript as a diagnostic issue, it also has epidemiologic relevance because stage distribution shapes survival patterns, treatment intensity, healthcare costs, and survivorship burden [10, 11]. Several contemporary reviews and registry-based analyses indicate that EOCRC patients are more likely than older patients to present with stage III or IV disease, reinforcing concerns that the rising incidence is compounded by delayed recognition and missed opportunities for earlier diagnosis [10, 11].

**Fig. 1:**
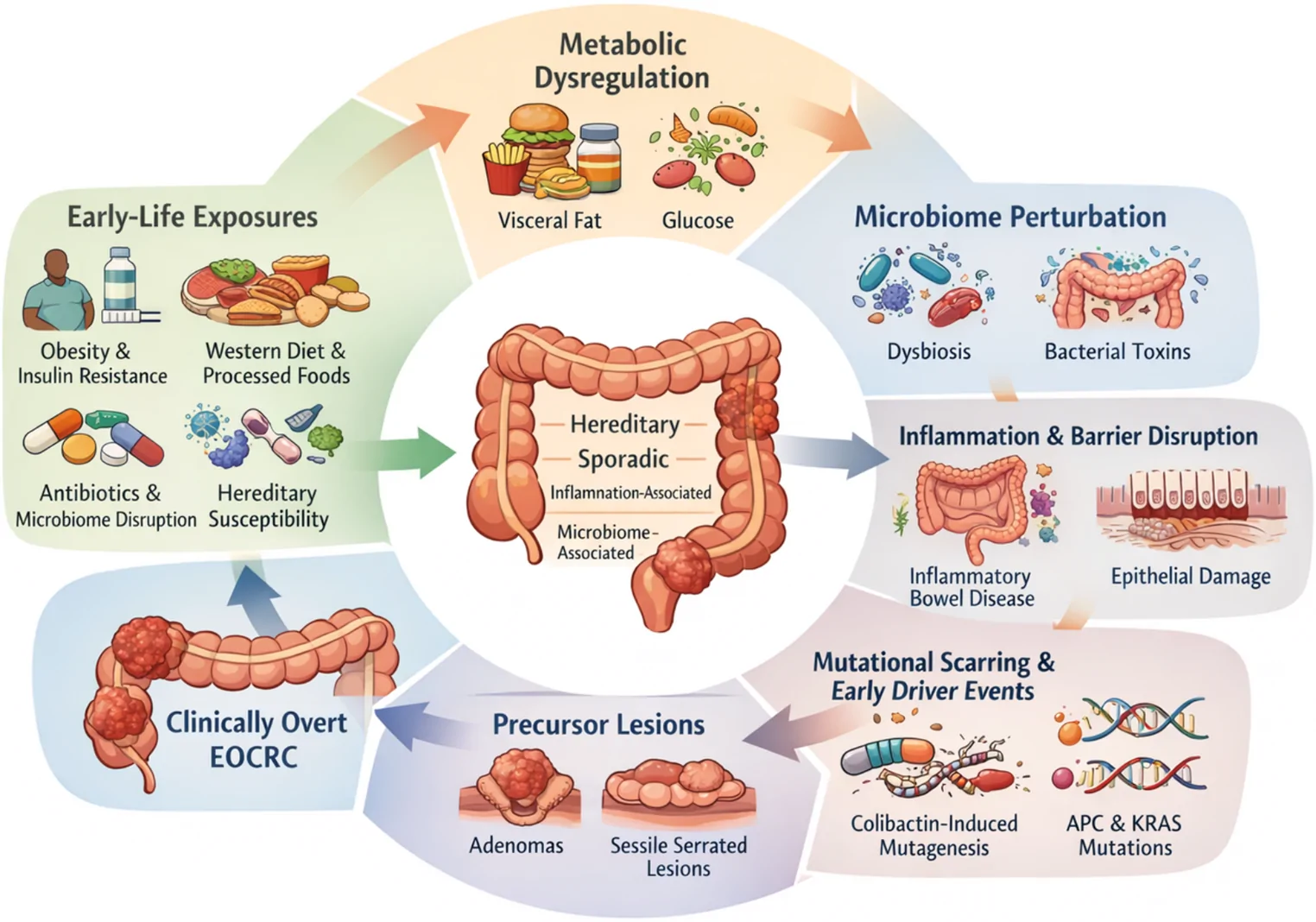
A life-course model of early onset colorectal cancer

Taken together, current epidemiologic evidence supports several key conclusions. First, EOCRC is a genuine and rising global phenomenon. Second, its increase appears to be driven disproportionately by distal colon and rectal cancers. Third, the strong birth-cohort signal argues for changing generational exposures as major contributors to risk. Finally, the combination of increasing incidence, younger age at onset, and frequent presentation at advanced stage makes EOCRC a major emerging challenge for cancer prevention, early detection, and clinical care [1, 2, 8–11]. These epidemiologic observations support a life-course model in which inherited susceptibility, metabolic dysfunction, early-life exposures, microbiome perturbation, inflammation, and mutational processes converge over time to promote clinically overt EOCRC (Fig. 1).

### Defining EOCRC: One Disease or Many?

EOCRC is most commonly defined as colorectal cancer diagnosed before the age of 50 years [12, 13]. This definition is clinically practical, widely used in epidemiologic studies, and historically aligned with older screening thresholds. However, it remains fundamentally operational rather than biologically validated [3, 12]. As a result, EOCRC is often treated as a single category for research and clinical discussion, even though accumulating data suggest that it represents a heterogeneous group of diseases rather than one uniform clinicopathologic entity [3, 12, 14].

The limitations of the age-based definition have become increasingly apparent in recent years. The cutoff of 50 years was established largely in relation to screening policy, not because of a clearly demonstrated molecular transition at that age [3, 12]. Since average-risk screening recommendations in the United States were lowered to begin at 45 years, the traditional boundary separating “early-onset” from “average-onset” disease has become even more conceptually unstable [3]. A 49-year-old patient diagnosed today may differ less biologically from a 51-year-old than from a 24-year-old, yet both younger individuals are still grouped together under the EOCRC label [3, 12]. This creates an important interpretive problem: EOCRC is a useful epidemiologic construct, but it should not automatically be assumed to be a biologically coherent subtype [12, 14].

A second major reason to reconsider EOCRC as a unified entity is that it encompasses both hereditary and apparently sporadic disease. Hereditary syndromes such as Lynch syndrome, familial adenomatous polyposis, MUTYH-associated polyposis, and rarer germline predisposition states account for an important fraction of younger-onset cases, especially among the very young and among patients with suggestive personal or family histories [12, 13, 15]. At the same time, most EOCRC cases are not explained by currently recognized hereditary syndromes. Contemporary reviews emphasize that the majority of EOCRC is sporadic, and recent germline sequencing work has shown that many patients with EOCRC, particularly those without classical hereditary phenotypes, do not harbor pathogenic variants in the major established colorectal cancer predisposition genes [13, 15, 16]. This distinction is crucial because hereditary EOCRC and sporadic EOCRC are unlikely to share identical biology, risk architecture, or prevention strategies [12, 15, 16].

The heterogeneity of EOCRC also extends to anatomic site. Population-based and review data consistently suggest that the recent increase in EOCRC is driven disproportionately by distal colon and rectal cancers [3, 12]. This matters not only epidemiologically, but also conceptually, because rectal cancer in young adults may differ from proximal colon cancer with respect to symptom profile, treatment burden, functional outcomes, and perhaps underlying biology [3, 12]. Accordingly, some investigators have argued that colon EOCRC and rectal EOCRC should not always be analyzed together without qualification [12].

Molecular data reinforce the view that EOCRC is not a single disease state. Available studies do not support one universal genomic signature that defines all EOCRC. Instead, current evidence points to substantial heterogeneity across tumors, with variation in mismatch repair status, chromosomal instability, epigenetic patterns, canonical driver alterations, and microbiome-associated mutational processes [14, 17]. A 2025 review of sporadic young-onset colorectal cancer concluded that most cases are sporadic and highlighted differences in somatic mutation patterns compared with later-onset disease, including reports of enrichment of certain alterations such as TP53 and CTNNB1 in some series, although the overall evidence remains inconsistent and cohort-dependent [14]. Likewise, more recent genomic analyses emphasize that EOCRC likely comprises multiple molecularly distinct subgroups rather than one reproducible “young-onset” profile [17].

Another source of heterogeneity lies in the age span included under the EOCRC umbrella. Patients diagnosed in their teens, twenties, thirties, and forties are often pooled in the same analyses, yet the underlying balance between hereditary predisposition, inflammatory bowel disease, metabolic exposures, reproductive factors, and cumulative environmental influences is unlikely to be identical across these subgroups [12, 13, 15]. The youngest patients may be enriched for strong germline predisposition, whereas cases arising closer to the age of 50 may be more heavily shaped by metabolic, dietary, and microbiome-related risk factors that overlap partially with later-onset disease [12–15]. This gradient is rarely captured by binary age cutoffs alone.

For these reasons, EOCRC is better viewed as an umbrella term that includes several partially overlapping phenotypes rather than as a single pathobiologic entity [12–14]. From a practical standpoint, at least four broad clinical-biological categories can be conceptualized: hereditary EOCRC, inflammation-associated EOCRC, metabolically or environmentally driven sporadic EOCRC, and molecularly distinctive EOCRC linked to emerging mechanisms such as microbiome-associated mutagenesis [12, 14–16]. These categories are not mutually exclusive, but they offer a more realistic framework for interpreting current literature and for designing future studies.

Recognizing the internal heterogeneity of EOCRC has important implications for both research and clinical care. It cautions against oversimplified comparisons between EOCRC and later-onset CRC, encourages more granular stratification by age, site, germline status, and molecular phenotype, and supports the need for tailored prevention and diagnostic strategies rather than a one-size-fits-all approach [3, 12–16]. Thus, the central question is not merely whether EOCRC differs from later-onset CRC, but which subsets of EOCRC differ, in what way, and through which biologic and clinical pathways [12, 14, 17].

### Etiology and Risk Architecture

The rapid rise of EOCRC has prompted intense interest in its underlying causes, yet the available evidence does not support a single unifying explanation [5, 8, 12]. Rather, EOCRC appears to arise through a multifactorial risk architecture shaped by the interaction of inherited susceptibility, metabolic dysfunction, diet and lifestyle, inflammatory states, microbial dysbiosis, and possibly early-life environmental exposures [8, 12, 26]. Importantly, the increase in EOCRC incidence across successive birth cohorts argues against heredity alone as the primary driver and instead supports a model in which contemporary generational exposures modify colorectal carcinogenesis in biologically susceptible individuals [5, 8]. Because EOCRC risk emerges from the convergence of established hereditary and inflammatory conditions, probable metabolic and lifestyle determinants, and still-evolving microbiome- and exposome-related mechanisms, the major risk domains should be interpreted as complementary rather than competing explanations. The current evidence landscape is summarized in Table 1, which distinguishes established, probable, emerging, and controversial factors according to their proposed mechanisms, clinical relevance, and remaining research gaps.

**Table 1:** Risk architecture of EOCRC: established, probable, emerging, and controversial factors

Risk domain	Specific factor	Strength of evidence	Proposed mechanism	Clinical relevance	Research gaps
Genetic / inherited predisposition	Hereditary syndromes (Lynch syndrome, FAP, MAP, polymerase proofreading syndromes)	Established [5, 11]	Germline defects in DNA repair, APC/WNT signaling, base-excision repair, or proofreading fidelity accelerate adenoma–carcinoma progression	High relevance for early diagnosis, surveillance, cascade testing, and treatment planning	Better delineation of less common predisposition genes and integration of germline findings with EOCRC phenotypes [1, 2]
Familial risk	Family history of CRC or advanced adenomas	Established [11, 18]	Shared germline susceptibility, shared environment, shared lifestyle/exposome	Directly actionable for earlier screening and hereditary risk capture	Need better family-history models that include polyp burden, age at diagnosis, and non-classical familial aggregation [2, 3]
Metabolic risk	Obesity / metabolic syndrome	Probable to Established [5, 19]	Hyperinsulinaemia, chronic low-grade inflammation, altered adipokine signaling, bile acid dysregulation, microbiome shifts	Major candidate driver of birth-cohort effect; supports life-course prevention strategies	Need stronger prospective life-course data and better causal disentangling from diet and inactivity [1, 4]
Metabolic risk	Diabetes / insulin resistance	Probable [5, 19]	Insulin–IGF signaling, pro-inflammatory milieu, altered energy metabolism, microbiome-mediated effects	Clinically relevant as part of metabolic-risk profiling	Need EOCRC-specific prospective estimates and mechanistic validation in younger populations [1, 4]
Lifestyle	Physical inactivity	Probable [19, 20]	Obesity promotion, insulin resistance, impaired immune-metabolic homeostasis, altered gut motility and inflammation	Modifiable risk domain; relevant to prevention counseling	Need more EOCRC-specific dose-response and interaction data with adiposity and diet [4, 5]
Diet / nutrition	Western diet / ultra-processed foods	Probable [5, 19]	Metabolic dysregulation, inflammatory signaling, reduced fiber exposure, altered bile acids, microbiome perturbation	High public-health relevance; plausible life-course contributor	Need more precise exposure quantification, harmonized dietary definitions, and prospective adolescent/early-adult data [1, 4]
Lifestyle	Alcohol	Probable [20, 21]	Acetaldehyde exposure, oxidative stress, folate perturbation, mucosal injury, microbiome alteration	Modifiable behavioral risk factor	More subtype-specific data needed, especially by colon vs. rectum and exposure intensity [5, 6]
Lifestyle	Smoking	Probable [20, 21]	Carcinogen exposure, oxidative DNA damage, inflammation, epigenetic effects, possible synergy with other exposures	Modifiable behavioral risk factor	Need better characterization of timing, cumulative exposure, and interaction with rectal-predominant EOCRC [5, 6]
Inflammatory conditions	Inflammatory bowel disease	Established [11, 22]	Chronic mucosal inflammation, epithelial turnover, oxidative damage, dysplasia-carcinoma sequence	Important for risk stratification and early surveillance in selected patients	Does not explain population-wide EOCRC rise; needs clearer integration into EOCRC subtype models [2, 7]
Medication / exposure-related	Antibiotics	Emerging / controversial [5, 23, 24]	Gut microbiome disruption, loss of colonization resistance, altered microbial metabolites and mucosal ecology	Potentially modifiable exposure, but not ready for risk-based recommendations	Human data are inconsistent; adulthood exposure may show weak or null associations, whereas cumulative or broad-spectrum exposure may matter more [8, 9]
Microbiome	Microbiome dysbiosis	Emerging [5]	Altered host–microbe interactions, immune dysregulation, epithelial barrier dysfunction, genotoxic/metabolic signaling	High conceptual importance; may become relevant for prevention and biomarker development	Still lacks standardized EOCRC-specific microbial signatures and causal validation across populations [1, 10]
Microbiome / mutagenesis	Colibactin-related exposure	Emerging [6]	Genotoxic injury from pks + *E. coli*, mutational scarring, early driver events including APC-related damage	One of the most compelling mechanistic hypotheses in EOCRC	Critical windows of exposure, prevalence across populations, and translational utility remain unresolved [10, 11]
Life-course / exposome	Early-life exposome	Speculative to emerging [5, 19, 25]	Prenatal, childhood, and adolescent exposures may shape microbiome maturation, metabolic programming, inflammation, and later carcinogenic susceptibility	Potentially central to birth-cohort effect and prevention thinking	Needs prospective birth-cohort or long-horizon exposome studies; currently strong conceptually, weak in direct causal proof [1, 4, 12]

A hereditary component remains an essential part of the EOCRC landscape. Pathogenic germline variants are more common in EOCRC than in later-onset CRC, and hereditary syndromes such as Lynch syndrome, familial adenomatous polyposis, MUTYH-associated polyposis, polymerase proofreading-associated polyposis, and other rare predisposition states account for a clinically significant subset of cases [12, 26]. However, hereditary disease explains only a minority of EOCRC overall. Recent genomic profiling studies and contemporary reviews consistently indicate that most EOCRC cases are sporadic, even when diagnosis occurs well before the traditional screening age [12, 26]. This distinction is critical, because it suggests that the rising incidence of EOCRC is unlikely to be explained by shifts in inherited cancer susceptibility at the population level and instead points toward broader environmental and host-related determinants [8, 26].

Among non-hereditary factors, the strongest current conceptual framework positions EOCRC as an emerging disease of metabolic dysregulation [5]. Cases of EOCRC have risen in parallel with increasing prevalence of obesity, insulin resistance, metabolic syndrome, and type 2 diabetes mellitus among children, adolescents, and younger adults [5]. A recent high-impact review argued that persistent exposure to metabolic dysfunction may alter intestinal physiology, bile acid signaling, inflammatory tone, insulin–IGF pathways, and gut microbial composition in ways that accelerate colorectal tumorigenesis at younger ages [5]. This life-course view is especially compelling because adiposity and metabolic disease begin decades before cancer diagnosis and may therefore help explain the birth-cohort effect observed in EOCRC epidemiology [5, 8].

The association between adiposity and EOCRC is supported by growing prospective evidence. A 2025 prospective investigation reported that adiposity, physical inactivity, smoking, and alcohol consumption were all associated with EOCRC risk, with adiposity showing a particularly notable signal in men [19]. These findings are consistent with broader literature implicating excess body weight and reduced physical activity as plausible contributors to early carcinogenesis in the colorectum [5, 19]. Still, causality is likely to be mediated through multiple interconnected mechanisms rather than simple body mass alone, including hyperinsulinaemia, chronic low-grade inflammation, altered gut permeability, and dysbiosis [5].

Diet is another major component of the EOCRC risk architecture, although the evidence is more heterogeneous than often presented. Western dietary patterns, high consumption of ultra-processed foods, red and processed meat intake, low fibre consumption, and sugar-sweetened beverages have all been proposed as contributors to EOCRC risk [5, 12]. Mechanistically, such exposures may promote obesity, insulin resistance, inflammatory signaling, and unfavorable shifts in the gut microbiome [5]. Although some specific dietary associations remain difficult to quantify consistently across studies, the overall pattern supports the idea that long-term nutritional exposures beginning early in life may be important in shaping EOCRC susceptibility [5, 12].

Alcohol and tobacco should also be considered established, if modest, contributors to EOCRC risk. A 2025 systematic review and meta-analysis found that alcohol use was associated with increased EOCRC risk, with a pooled odds ratio of 1.39, and smoking showed a similar pooled odds ratio of 1.39 [20]. The same analysis also identified a positive dose-response relationship for alcohol consumption [20]. These findings do not suggest that alcohol and smoking are the dominant drivers of EOCRC, but they do reinforce the broader conclusion that younger-onset disease shares at least part of its etiologic architecture with conventional colorectal carcinogenesis while potentially being amplified by earlier and more sustained exposure across the life course [12, 20].

Chronic inflammatory conditions, particularly inflammatory bowel disease (IBD), represent another important but quantitatively insufficient explanation for the rise in EOCRC [12]. Longstanding mucosal inflammation is a well-established risk factor for colorectal neoplasia, and young patients with ulcerative colitis or Crohn’s colitis may develop CRC substantially earlier than the general population [12]. However, IBD prevalence alone is unlikely to account for the widespread population-level increase in EOCRC incidence [5, 12]. Its significance lies more in defining one biologically distinct subgroup of EOCRC than in explaining the overall epidemiologic phenomenon.

Over the past few years, the gut microbiome has moved from a speculative factor to one of the most scientifically compelling areas in EOCRC research [6, 22]. A growing body of evidence suggests that microbial dysbiosis may contribute to EOCRC through interactions with host immunity, epithelial barrier function, inflammation, and direct genotoxicity [22]. Among the most influential recent findings is the identification of mutational signatures linked to colibactin-producing bacteria. In a landmark 2025 genomic study, mutational patterns associated with colibactin exposure were found to be enriched in younger patients with colorectal cancer, being 3.3-fold more common in tumors diagnosed before age 40 than in tumors diagnosed after age 70 [6]. These signatures were also linked to early APC driver events, supporting the hypothesis that bacterial genotoxins may imprint the genome long before cancer becomes clinically apparent [6]. This finding is particularly important because it provides a mechanistic bridge between early-life microbial exposure and later malignant transformation.

Microbiome-related hypotheses also help connect several other proposed EOCRC risk factors into a coherent biologic model. Metabolic dysfunction, dietary westernisation, antibiotic exposure, altered bile acid metabolism, and low-grade inflammation can all reshape microbial ecology and microbial metabolite production [5, 22]. In this framework, EOCRC is not caused by a single bacterium or exposure, but may emerge from a disturbed host–microbiome ecosystem that promotes mutagenesis, proliferative signaling, and failure of mucosal homeostasis over time [5, 6, 22]. This model remains incomplete, but it is increasingly plausible and intellectually productive.

An additional and highly relevant concept is the early-life exposome. Because the strongest epidemiologic signal in EOCRC is a birth-cohort effect, many investigators now argue that carcinogenic influences likely begin much earlier than symptom onset or diagnosis [5, 6, 8]. Prenatal factors, childhood obesity, adolescent diet, antibiotic use, microbiome maturation, environmental toxicants, and cumulative metabolic injury have all been proposed as components of a life-course risk model [5, 8, 22]. Although direct evidence for many of these exposures remains preliminary, this framework is attractive because it explains how EOCRC incidence could increase rapidly across generations without requiring major changes in inherited susceptibility [5, 8].

Taken together, current evidence supports a layered model of EOCRC causation. Hereditary syndromes remain essential to identify but explain only a minority of cases [12, 26]. The broader rise in EOCRC is more plausibly linked to the interaction of metabolic dysfunction, dietary and behavioral exposures, chronic inflammation, and microbiome-mediated biologic effects operating across the life course [5, 6, 8, 19, 20]. The most defensible conclusion at present is therefore not that one cause has been found, but that EOCRC reflects a convergence of multiple risk domains whose relative importance likely differs across patients and subtypes [5, 8, 12, 22]. This heterogeneity should shape both future research and clinical strategies for prevention, early detection, and risk stratification.

### Biology and Molecular Pathogenesis

The molecular biology of EOCRC is increasingly recognized as complex, heterogeneous, and only partially distinct from that of later-onset colorectal cancer (LOCRC) [12, 14, 17]. Although EOCRC is often discussed as a separate clinical entity, current evidence does not support the existence of one universal molecular signature that defines all tumours arising before the age of 50 years [12, 14, 17, 27]. Rather, EOCRC appears to encompass multiple biologic subgroups shaped by varying contributions of germline predisposition, mismatch repair deficiency, chromosomal instability, epigenetic alteration, oncogenic pathway activation, and microbiome-associated mutagenesis [12, 14, 17, 27].

A key principle in interpreting this literature is that EOCRC shares much of the canonical molecular framework of conventional colorectal carcinogenesis. Core pathways involving APC/WNT dysregulation, RAS–RAF–MAPK activation, TP53 loss, mismatch repair deficiency, and chromosomal instability remain relevant in younger patients, and many EOCRC tumours still follow recognizable adenoma–carcinoma or serrated-pathway biology [12, 17, 28]. However, the relative distribution, timing, and interaction of these mechanisms may differ across younger age groups and tumour subsites, suggesting that EOCRC is not simply a younger chronologic version of the same disease [12, 14, 17].

One of the most important distinctions within EOCRC is the separation between mismatch repair-deficient and mismatch repair-proficient disease. A subset of EOCRC is driven by Lynch syndrome and related germline defects in DNA mismatch repair genes, resulting in microsatellite instability-high (MSI-H) or mismatch repair-deficient (dMMR) tumours [12, 17]. These tumours are biologically and clinically important because they often arise at younger ages, may carry a marked familial component, and have implications for immunotherapy responsiveness, cascade testing, and surveillance of relatives [17]. At the same time, most EOCRC is mismatch repair-proficient and microsatellite stable, emphasizing again that hereditary hypermutated disease explains only a fraction of the younger-onset burden [14, 17].

Beyond hereditary dMMR tumours, the overall somatic genomic landscape of sporadic EOCRC appears broadly similar to that of later-onset CRC, but with notable shifts in frequency and pattern rather than wholesale replacement by a new biology [14, 27]. A recent comprehensive genomic analysis of sporadic EOCRC found that the overall landscape largely overlaps with that of later-onset CRC, yet reported enrichment of alterations such as NOTCH1, FBXW7, PIK3CA, FGFR3, and certain KRAS G12 variants in EOCRC compared with later-onset disease [27]. Other recent reviews also describe possible enrichment of TP53 and CTNNB1 alterations in some EOCRC cohorts, although these findings are not consistent across all studies and likely depend on cohort composition, tumour location, and selection bias [14, 17]. Thus, the most defensible conclusion is that EOCRC may show quantitative shifts in somatic architecture rather than a single reproducible set of pathognomonic mutations [14, 17, 27].

Chromosomal instability remains a major molecular backbone of EOCRC, especially in microsatellite-stable tumours [12, 17]. Many younger-onset tumours exhibit the canonical pattern of APC, KRAS, and TP53 disruption associated with conventional colorectal tumorigenesis [12, 28]. However, recent large-scale genomic work has suggested that early-onset disease may be enriched for certain molecular configurations and rare subgroups not fully appreciated in earlier, smaller cohorts [28]. In a 2024 genomic analysis of more than 2,000 colorectal cancers, investigators identified novel molecular features associated with early-onset disease and tumour location, further reinforcing the idea that age interacts with colorectal cancer biology in a nuanced, subtype-dependent manner rather than through a single binary mechanism [28].

Epigenetic regulation is another important but still incompletely resolved area in EOCRC biology. Several studies suggest that methylation patterns in EOCRC may differ from those seen in later-onset disease, although results are not entirely uniform [12, 14, 17]. In particular, some younger tumors appear less likely to follow the classic serrated/CIMP-high pathway typical of a subset of older proximal colon cancers, whereas others show distinct epigenetic changes that may reflect accelerated tumorigenesis rather than simple biologic immaturity [12, 17]. At present, however, evidence remains insufficient to define a single EOCRC-specific epigenetic phenotype, and this remains an active area for future research [14].

Transcriptomic and microenvironmental heterogeneity add another layer of complexity. Emerging data suggest that poor-prognosis young-onset colorectal cancer may be enriched for mesenchymal-like or stromal-rich phenotypes in at least some cohorts, with consequences for invasion, immune contexture, and treatment response [29]. These observations are provocative because they raise the possibility that a subset of EOCRC may be defined less by classic single-gene alterations and more by ecosystem-level features involving epithelial–stromal interaction, extracellular matrix remodelling, and immune suppression [29]. However, these findings are still evolving and should be interpreted cautiously until validated across diverse populations.

Among the most influential recent advances in EOCRC biology is the growing evidence linking microbiome-derived genotoxicity to early colorectal tumorigenesis [6, 30]. In a landmark 2025 Nature study, mutational signatures associated with exposure to colibactin-producing bacteria were found to be 3.3-fold more frequent in colorectal cancers diagnosed before the age of 40 than in tumours diagnosed after the age of 70 [6]. Colibactin-associated mutational processes were further linked to APC driver alterations, with the ID18 signature accounting for approximately 25% of APC driver indels in colibactin-positive cases [6]. These findings are highly consequential because they provide a mechanistic framework in which early-life microbial exposure can leave a durable genomic imprint that later contributes to malignant transformation [6]. In other words, at least some EOCRC may reflect mutagenic events-initiated years or even decades before diagnosis.

Despite these advances, an important caution remains: current molecular data do not justify treating EOCRC as a biologically uniform class [14, 17, 27]. Many studies remain retrospective, definitions of EOCRC are inconsistent, very young patients are often pooled with those in their late forties, and cohort composition varies substantially by hereditary burden, tumour site, and ethnicity [12, 14, 17]. As a result, some reported molecular “differences” may actually reflect hidden differences in case mix rather than truly age-specific biology [14, 17]. Future work will therefore need to move beyond simple EOCRC-versus-LOCRC comparisons and instead define molecularly and clinically meaningful EOCRC subgroups.

Overall, the current state of evidence supports a nuanced view of EOCRC molecular pathogenesis. EOCRC shares the fundamental molecular architecture of colorectal cancer, but within that shared framework there appear to be age-associated shifts in pathway usage, mutational processes, and tumour ecosystem features [6, 12, 14, 17, 27–29]. The strongest emerging signals include molecular heterogeneity within EOCRC, the distinction between hereditary dMMR and largely sporadic microsatellite-stable disease, and the growing evidence that microbiome-associated mutagenesis—particularly colibactin-related damage—may contribute to early driver events in a subset of tumours [6, 12, 14, 17, 27, 30]. These insights do not yet define one EOCRC biology, but they do make clear that the disease cannot be fully understood through age alone.

Several clinicopathologic and molecular differences between early-onset colorectal cancer (EOCRC) and later-onset colorectal cancer (LOCRC) have been described. These differences involve tumour location, stage at presentation, prevalence of hereditary syndromes, and selected molecular features, although many findings remain heterogeneous across cohorts. A summary of the most consistently reported distinctions is presented in Table 2.

**Table 2:** Molecular and clinicopathologic features of EOCRC versus later-onset CRC

Feature	EOCRC	LOCRC	Interpretation / comment
Age at diagnosis	< 50 years	Usually ≥ 50 years	Operational distinction used in most studies; clinically practical, but biologically imperfect because the cutoff is screening-based rather than mechanistically defined [3, 12]
Family history	More frequent than in LOCRC, but absent in many cases	Less frequent overall, though still relevant	The association is consistent, but most EOCRC is not explained by family history alone; absence of family history does not exclude clinically meaningful risk [3, 12, 17]
Hereditary syndrome burden	Higher	Lower	One of the most consistent differences. Lynch syndrome and other germline predisposition syndromes are enriched in EOCRC, but still account for only a minority of all EOCRC cases [12, 13, 17]
Tumor site	More often distal colon and rectum	More mixed; proportionally more proximal disease with age	The distal/rectal predominance in EOCRC is one of the most reproducible clinicopathologic observations [1, 12]
Stage at presentation	More often stage III/IV	More often diagnosed through screening or earlier work-up	Consistent across many cohorts and likely linked in part to diagnostic delay and lack of screening below routine age thresholds [12, 31]
dMMR / MSI-H frequency	Higher than in average sporadic LOCRC when hereditary cases are included; variable in sporadic EOCRC	Lower overall in unselected younger cohorts, but MSI-H/dMMR also present in older sporadic and Lynch-related CRC	Interpretation depends heavily on cohort composition. Enrichment is largely driven by hereditary/Lynch-associated EOCRC; sporadic EOCRC is often MSS/pMMR [12, 14, 17]
Common somatic alterations	Broad overlap with conventional CRC; possible enrichment of selected alterations in some cohorts (e.g., TP53, CTNNB1, NOTCH1, FBXW7, PIK3CA, FGFR3, certain KRAS variants)	Canonical APC/KRAS/TP53 backbone with age- and site-dependent variation	Data are not fully consistent. The main message is quantitative shift and heterogeneity rather than one unique EOCRC genomic signature [14, 17, 27]
Epigenetic features	Possibly less classic age-related serrated/CIMP-high pattern in some cohorts; epigenetic landscape still heterogeneous	More established representation of age-associated methylation patterns and serrated/CIMP-high pathways in selected subgroups	Evidence is suggestive but not definitive. No single EOCRC-specific epigenetic phenotype has been validated [12, 14, 17]
Microbiome-related hypotheses	Stronger emphasis on dysbiosis, early-life microbial perturbation, and colibactin-associated mutational scarring	Microbiome also relevant, but early-life exposure model is less central conceptually	One of the most exciting emerging distinctions. Mechanistic support is growing, especially for colibactin-related mutational signatures, but clinical translation remains early [6, 30]
Symptom burden	Rectal bleeding, abdominal pain, altered bowel habits, anemia, and weight loss are common; diagnosis often symptom-driven	More often screen-detected or investigated earlier in screening-eligible populations	Symptoms are not necessarily subtler in EOCRC; the key difference is that they are more often initially underestimated or misattributed [1, 31]
Treatment intensity	Often more aggressive multimodality treatment due to younger age, better performance status, and advanced stage at diagnosis	Often guided by comorbidity burden and standard stage-based care	Younger patients are more likely to receive intensive treatment, but evidence does not support escalation by age alone; the issue is careful selection, not reflex intensification [13, 32, 33]
Survivorship burden	Greater long-term burden related to fertility, sexual health, work disruption, financial toxicity, parenting, and long-term function	Survivorship burden remains substantial, but average impact on reproductive and occupational life-course issues is lower	One of the clearest real-world differences. EOCRC survivorship is shaped by timing of disease in the life course as much as by tumor biology [13, 33, 34]

### Clinical Presentation and Diagnostic Delay

The clinical presentation of EOCRC is paradoxical: many patients develop recognizable alarm symptoms, yet diagnosis is frequently delayed [4, 31]. This pattern is one of the defining clinical features of EOCRC and helps explain why younger adults are often diagnosed with more advanced disease than their older counterparts [4, 11]. Because most individuals under 50 years are outside routine population-based screening pathways, diagnosis depends heavily on symptom recognition, appropriate help-seeking, timely clinical suspicion, and prompt referral for colonoscopic evaluation [4, 10]. When any of these steps fail, the consequence is often a prolonged diagnostic interval.

The symptom profile of EOCRC is now better characterized than it was only a few years ago. In a 2024 systematic review and meta-analysis including 81 studies and more than 24.9 million individuals younger than 50 years, the most common presenting features were haematochezia, reported in 45% of cases, abdominal pain in 40%, and altered bowel habits in 27% [31]. The same analysis found that haematochezia, abdominal pain, and anaemia were associated with higher EOCRC likelihood, with haematochezia showing the strongest association, ranging from approximately 5-fold to 54-fold across included studies [31]. Other contemporary reviews likewise describe rectal bleeding, abdominal pain, diarrhoea or constipation, iron-deficiency anaemia, weight loss, and reduced appetite as the dominant symptom constellation in EOCRC [11]. In practice, therefore, EOCRC is not usually “silent”; the greater problem is that its symptoms are common, nonspecific, and too often attributed to benign conditions.

This mismatch between symptom presence and diagnostic action is clinically critical. The 2026 Annual Review of Medicine article on EOCRC emphasizes that, despite overt symptoms, younger patients continue to experience prolonged diagnostic delays because the pathway to diagnosis is hindered by both patient-level and provider-level barriers [4]. Younger adults may normalize rectal bleeding, self-manage bowel symptoms, or delay presentation because cancer is not perceived as likely at their age [4]. At the clinician level, symptoms may initially be attributed to haemorrhoids, irritable bowel syndrome, stress-related gastrointestinal disturbance, postpartum changes, or other benign explanations, particularly in the absence of a strong family history [4]. This combination of low pretest suspicion and symptom nonspecificity creates a setting in which EOCRC can remain unrecognized for months.

The magnitude of this delay is not trivial. The 2024 JAMA Network Open meta-analysis found that the interval from sign or symptom presentation to EOCRC diagnosis was commonly in the range of 4 to 6 months, with a mean of 6.4 months and a median of 4 months across included studies [31]. A recent review similarly noted average delays of four to six months, with some reports describing substantially longer intervals [11]. Importantly, these delays often occur even in the presence of red-flag features that, in an older adult, would usually trigger expedited investigation [4, 11, 31]. In other words, the problem is not simply late symptom onset, but a systematic failure to respond to symptoms with appropriate urgency in younger patients.

This has direct consequences for stage at diagnosis. A 2025 British Journal of Cancer analysis noted that EOCRC is typically diagnosed at a more advanced stage than later-onset CRC and explicitly linked this pattern to the lack of screening and reliance on symptomatic presentation in younger individuals [10]. The same paper states that, in patients under 50 years, diagnosis depends on symptoms because screening is generally absent, and that the possibility of cancer is rarely considered early, contributing to delayed diagnosis and advanced-stage presentation [10]. These observations are concordant with broader reviews reporting a higher likelihood of stage III or IV disease among EOCRC patients [4, 10, 11].

A useful way to conceptualize this problem is as a sequence of “missed opportunities” along the diagnostic pathway rather than a single delay event. The Annual Review article frames EOCRC detection around several stages: symptom appraisal by the patient, first presentation to care, clinician interpretation, referral decisions, and completion of definitive testing [4]. Delay can occur at any step, and multiple small delays may accumulate before diagnosis [4]. This framework is especially valuable because it shifts the discussion away from blaming either patients or clinicians alone and instead highlights the structural weaknesses of an age-centered diagnostic model.

Given that symptomatic pathways now carry so much of the diagnostic burden in EOCRC, interest has grown in using the faecal immunochemical test (FIT) as a triage tool for younger symptomatic patients. In the 2025 South West England study, 38,117 symptomatic individuals aged 18–49 years underwent FIT in primary care, and a positive threshold of 10 µg Hb/g faeces was used [10]. The study was designed in response to the reality that, in the absence of screening for those under 50 years, diagnosis relies on symptoms and that evidence for FIT performance in this age group had been limited [10]. This work supports the potential utility of FIT in the diagnostic pathway for younger adults, although it does not replace the need for careful clinical judgment or definitive endoscopic evaluation when suspicion remains high [10]. In other words, FIT may help triage, but it should not become a convenient excuse to underinvestigate persistent red-flag symptoms.

Prospective data are beginning to confirm that these longer diagnostic intervals are real and not merely an artefact of retrospective recall. A 2025 prospective cohort study from New Zealand specifically examined the pathway to diagnosis in EOCRC and was motivated by prior evidence suggesting that younger patients experience longer diagnostic intervals than older adults, potentially contributing to poorer outcomes [35]. Although the broader field still needs more prospective and health-system-level studies, the direction of the evidence is now consistent: younger adults with CRC often move through slower and less direct diagnostic pathways than older patients [4, 35].

Taken together, the clinical picture of EOCRC is now clear. The disease commonly presents with recognizable gastrointestinal warning signs, particularly rectal bleeding, abdominal pain, altered bowel habits, and anaemia [11, 31]. However, because younger adults are generally unscreened and because cancer is often not initially suspected in this age group, these symptoms too often fail to trigger rapid investigation [4, 10]. The result is a prolonged diagnostic interval, frequent presentation with advanced-stage disease, and avoidable loss of opportunity for earlier treatment [4, 10, 11, 31, 35]. For this reason, diagnostic delay should be regarded not as a secondary issue in EOCRC, but as one of its central clinical problems.

### Screening, Early Detection, and Prevention

The rise of EOCRC has exposed a central limitation of current prevention strategies: traditional colorectal cancer screening models were designed around the epidemiology of older adults and are therefore only partially suited to the modern EOCRC landscape [3, 36, 37]. Lowering the screening age from 50 to 45 years was an important public health response, but it should be viewed as a necessary adjustment rather than a complete solution [3, 36, 37]. A substantial proportion of EOCRC still occurs before the age of 45 years, meaning that many patients remain outside average-risk screening pathways even after guideline revision [3, 36].

Current U.S. recommendations are now well established. The US Preventive Services Task Force recommends colorectal cancer screening for average-risk adults aged 45 to 75 years, with individualized decision-making for those aged 76 to 85 years [3]. The CDC echoes this framework and also emphasizes that people at increased risk, including those with a strong family history, hereditary cancer syndromes, or inflammatory bowel disease, may need earlier and more intensive screening than the average-risk population [36]. These recommendations are clinically important in EOCRC because they formalize the shift away from the historical age threshold of 50 years, but they also underscore that prevention must now be understood in both age-based and risk-based terms.

The practical effect of lowering the screening age has been measurable, but incomplete. A 2025 cohort study found that facility-based colorectal cancer screening among adults aged 45–49 years increased nearly tenfold after the USPSTF guideline change, far exceeding the growth observed in older age groups during the same period [37]. At the same time, newer population-level analyses indicate that uptake in newly eligible adults remains suboptimal. A 2025 study of screening among adults aged 45–49 years reported that screening rates in 2022 were still low and showed important disparities by sociodemographic factors and by access to endoscopic services, suggesting that guideline change alone does not ensure effective implementation [38]. In practice, then, the problem is no longer just whether to start at 45, but how to achieve equitable and meaningful uptake once eligibility begins.

Even if screening from age 45 is fully implemented, however, it will not capture the whole EOCRC burden. Much of the current policy discussion risks implying that the screening-age shift solves the problem of younger-onset disease, when in fact it addresses only the upper slice of the EOCRC age spectrum [32, 3, 4]. The remaining challenge is the patient younger than 45 years who is not eligible for routine screening and must therefore be identified through family history, hereditary risk recognition, symptom-triggered investigation, or future precision-prevention tools [4]. This is why the EOCRC field increasingly distinguishes between screening, which applies to asymptomatic individuals, and early detection, which in many younger adults must rely on more targeted, risk-adapted strategies.

Family history and inherited susceptibility remain the most actionable current gateways to earlier prevention. Contemporary EOCRC-focused reviews emphasize that careful family-history assessment, systematic identification of hereditary cancer syndromes, and appropriate germline evaluation are among the few established ways to move screening and surveillance earlier than age-based average-risk recommendations [4]. This has practical implications not only for individuals with Lynch syndrome, familial adenomatous polyposis, or other recognized predisposition states, but also for patients with less formally defined familial clustering who may still benefit from accelerated colonoscopic strategies [4, 36]. In this sense, improving hereditary risk capture is not a peripheral issue in EOCRC prevention; it is one of the most immediately actionable interventions available today.

At the same time, screening policy cannot be separated from symptomatic detection. Because many EOCRC cases arise before screening eligibility, early diagnosis in younger adults still depends heavily on whether warning signs are recognized and investigated promptly [4]. Recent work has therefore placed increasing emphasis on the faecal immunochemical test (FIT) as a triage tool for symptomatic younger adults in primary care. The 2025 British Journal of Cancer analysis of patients aged 18–49 years specifically addressed this gap, noting that in the absence of screening in younger patients, diagnosis relies on symptomatic presentation and that FIT may help prioritize urgent referral in this group [10]. FIT should not be confused with population screening in this context; rather, it may serve as a pragmatic bridge between symptoms and definitive endoscopic evaluation when age-based screening does not apply.

These limitations have driven growing interest in risk-stratified screening. A 2025 review on risk-based colorectal cancer screening argues that tailoring screening intensity according to age, sex, race or ethnicity, lifestyle factors, genetic predisposition, and prior screening results could improve efficiency, cost-effectiveness, and resource allocation compared with uniform age-based strategies [39]. For EOCRC, the appeal of this model is obvious: it offers a conceptual route to identify people who are biologically or clinically at higher risk before they reach a conventional screening threshold [39]. However, the same review also highlights major barriers, including limited validation of risk tools, uneven data quality, health-system constraints, and ethical concerns around implementation [39]. Thus, risk-stratified screening is promising, but not yet mature enough to replace current standard approaches.

Beyond classical risk models, the field is beginning to move toward precision prevention. Educational and translational reviews from 2025 describe EOCRC as a setting in which family history, germline findings, lifestyle risk domains, structured electronic health records, and emerging molecular markers may eventually be integrated into more personalized prevention frameworks [4, 40]. Early machine-learning work using structured EHR data in individuals younger than 45 years has already explored the feasibility of identifying EOCRC-related risk patterns before diagnosis, although this approach remains investigational and not yet ready for routine clinical use [40]. Still, these developments signal a broader shift in thinking: the future of EOCRC prevention is unlikely to rely on age alone.

Prevention must also be understood across the life course, not merely at the moment screening begins. Emerging reviews in EOCRC increasingly argue that prevention may need to start conceptually decades before diagnosis, through control of obesity, metabolic dysfunction, poor diet, sedentary behavior, smoking, and alcohol exposure, as well as through improved recognition of high-risk inflammatory and hereditary states [4]. This does not mean that lifestyle modification alone can reverse EOCRC incidence trends, but it does mean that a credible prevention framework must extend beyond colonoscopy schedules and incorporate upstream determinants of risk. In that sense, screening is only one component of EOCRC prevention, not its entirety.

### Treatment Considerations

At present, the treatment of EOCRC is not defined by wholly separate age-specific oncologic algorithms. In routine practice, surgery, systemic therapy, radiotherapy for rectal cancer, and biomarker-directed treatment are applied primarily according to stage, tumour location, resectability, and molecular profile rather than age alone [9, 13]. However, this does not mean that EOCRC should be managed identically to later-onset colorectal cancer in practical terms. The more accurate view is that the antitumor backbone remains largely shared, whereas the broader therapeutic framework often needs to be more deliberate and individualized in younger patients because of advanced stage at diagnosis, rectal-cancer predominance, greater likelihood of hereditary susceptibility, longer survivorship horizon, and the much greater long-term burden of functional, reproductive, occupational, and psychosocial consequences [9, 13].

A central clinical question is whether EOCRC carries intrinsically worse outcomes or whether it appears more threatening largely because it is diagnosed later. Current evidence supports a nuanced interpretation. A recent systematic review and meta-analysis found that EOCRC is more often diagnosed at an advanced stage, yet this is not consistently associated with poorer cancer-specific survival overall; in fact, EOCRC may show better overall survival in some analyses, likely reflecting lower competing mortality and better tolerance of intensive treatment in younger adults [32]. At the same time, this apparent advantage is not uniform across all subgroups. Available data suggest that rectal EOCRC may carry less favourable disease-free survival than later-onset rectal cancer, and metastatic EOCRC may behave more aggressively, or at least follow a shorter disease-control trajectory, in selected patients [32, 41]. Accordingly, age itself should not be treated as a surrogate either for favourable or unfavourable prognosis; stage, biology, site, and therapeutic context remain the dominant determinants of outcome [32, 41].

Surgery remains the cornerstone of curative treatment for localized EOCRC [9, 13]. In colon cancer, operative principles are fundamentally the same as in older adults. In rectal cancer, however, treatment decisions have especially profound long-term consequences in younger patients. Because EOCRC disproportionately affects the distal colon and rectum, many younger adults face total mesorectal excision, pelvic radiotherapy, temporary or permanent stomas, and chronic bowel, urinary, and sexual dysfunction at a much earlier stage of life [13, 42]. This is precisely why current rectal cancer management has moved toward more response-adapted and function-conscious strategies. Recent NCCN Insights for rectal cancer incorporated updated total neoadjuvant therapy approaches and recognized watch-and-wait as a nonoperative option for selected patients with a clinical complete response after neoadjuvant treatment [33]. For younger adults, this is particularly relevant because organ preservation, when oncologically sound, may reduce the lifelong burden of pelvic-treatment morbidity without compromising cancer control in carefully selected cases [33, 42].

Systemic therapy in EOCRC also follows standard disease-based principles, but several practical differences are important. Younger patients often have better performance status and are therefore more likely to receive intensive multi-agent chemotherapy, multimodality therapy, and metastasis-directed strategies [13, 41]. However, younger age should not automatically trigger therapeutic escalation. Contemporary EOCRC reviews consistently emphasize that the relevant question is not simply whether a patient can tolerate more treatment, but whether the expected gain justifies the price in terms of neuropathy, infertility, sexual dysfunction, bowel dysfunction, premature menopause, financial toxicity, and long-term impairment in quality of life [9, 13]. In other words, EOCRC management should be more thoughtful, not reflexively more aggressive.

This principle is well aligned with current NCCN guidance for colon cancer, which remains strongly stage- and biomarker-driven, especially in metastatic disease [33]. Recent NCCN-based updates emphasize treatment selection according to mismatch repair status and other clinically actionable biomarkers, including RAS, BRAF, and HER2, with broader genomic profiling increasingly relevant in selected settings [33]. In this respect, EOCRC is a setting in which comprehensive molecular workup is particularly important. Younger patients are enriched for hereditary syndromes and may also derive greater therapeutic and familial benefit from early identification of dMMR/MSI-H status and other targetable alterations [13]. NCCN materials also indicate that tumours with dMMR/MSI-H should prompt evaluation for Lynch syndrome, reinforcing the integration of treatment planning with hereditary risk assessment [43].

The hereditary dimension of treatment planning is especially important in EOCRC. Current NCCN-linked recommendations support germline multigene panel testing for all patients diagnosed with colorectal cancer before the age of 50 years [43–45]. This has direct therapeutic relevance, because identification of Lynch syndrome or other germline predisposition syndromes may influence systemic therapy discussions, surveillance recommendations, surgical planning in selected contexts, and cascade testing for family members [13, 43, 45]. In younger patients, therefore, germline assessment should be viewed not as an optional adjunct to oncology care, but as part of standard-quality management.

These considerations are amplified further in metastatic EOCRC. Recent studies suggest that some younger patients with metastatic disease may follow a particularly challenging course despite good baseline fitness, whereas others achieve prolonged survival when treated in multidisciplinary pathways that include resection or ablation of metastatic disease whenever feasible [41]. This reinforces two complementary principles: first, metastatic EOCRC should be evaluated early by multidisciplinary teams rather than being approached through a purely palliative lens from the outset; second, enthusiasm for aggressive treatment must still be tempered by biologic realism and by the patient’s long-term priorities [42]. The goal is not indiscriminate intensification, but precision selection.

Rectal cancer deserves separate emphasis because it concentrates many of the most distinctive treatment dilemmas of EOCRC. Functional outcomes after rectal cancer therapy remain a major issue even in older adults, but their impact is often magnified in younger patients who may live for decades with the consequences of treatment [42]. Recent literature suggests that watch-and-wait and other organ-preserving strategies may improve quality of life and selected functional outcomes in properly chosen patients after neoadjuvant therapy [33, 42]. For EOCRC, this matters greatly: treatment decisions made at age 30 or 40 may shape continence, sexual health, reproductive planning, body image, and employability over the remainder of adult life. As a result, oncologic adequacy remains the first priority, but functional preservation must be taken far more seriously than in a simplistic “young patients tolerate more” framework.

Taken together, the most defensible principle is not that EOCRC requires a completely separate treatment paradigm, but that it requires a more comprehensive and future-oriented one. The therapeutic backbone remains stage-, site-, and biomarker-based, in accordance with current colorectal cancer standards and NCCN guidance [33, 43]. What should differ is the consistency of germline and tumour profiling, the seriousness with which organ-preserving and metastasis-directed options are considered, and the extent to which long-term functional and survivorship consequences are integrated into up-front decision-making [13, 33, 42–45]. In this sense, EOCRC does not demand a different oncology textbook, but it does require a different clinical posture.

### Survivorship and Age-Specific Burden

Survivorship in EOCRC extends far beyond oncologic control and should be regarded as a central component of care rather than a downstream afterthought [46–48]. Because EOCRC affects patients during the most socially, professionally, and reproductively active decades of life, its consequences often unfold across a much longer time horizon than in later-onset disease [46, 47]. As a result, even when cancer outcomes are favourable, younger survivors may experience a disproportionate long-term burden related to fertility, sexual function, employment, financial hardship, body image, interpersonal relationships, and fear of recurrence [34, 46–49].

One of the most distinctive survivorship issues in EOCRC is reproductive health. Recent reviews emphasize that fertility concerns in young-onset colorectal cancer are among the most important quality-of-life priorities for patients and may rank just after survival itself in perceived importance [47, 48]. Yet these concerns are still inconsistently addressed in routine care [34, 47, 48]. A 2025 review focused on sexual and reproductive health in EOCRC concluded that treatment-related gonadal toxicity, fertility decline, and pregnancy-related issues remain underrecognized, despite their potentially major impact on long-term wellbeing [34]. Similarly, a 2024 review on fertility in young-onset colorectal cancer highlighted that surgery, chemotherapy, pelvic radiotherapy, and ostomy-related treatment pathways may all affect reproductive potential directly or indirectly, making fertility preservation counselling a critical part of pre-treatment planning whenever feasible [48]. These observations are particularly relevant in rectal cancer, where pelvic treatment may affect ovarian reserve, uterine exposure, ejaculation, erectile function, and future pregnancy outcomes [34, 48].

Sexual health is closely linked to fertility but should be considered a distinct survivorship domain. Available evidence suggests that sexual dysfunction is common after EOCRC treatment and remains insufficiently discussed in clinical encounters [34, 47, 49]. A 2025 study of EOCRC patients reported that fertility discussions occur more often than sexuality discussions, despite substantial sexual side effects in both women and men [49]. The same study highlighted that women may experience more libido reduction, vaginal dryness, and sexual pain, while pelvic treatment in rectal cancer may also affect sexual functioning more broadly [49]. This imbalance is clinically important: preserving reproductive options is not the same as preserving sexual wellbeing, and survivorship care that addresses only fertility leaves a major gap in quality-of-life support [34, 49].

Psychological and psychosocial burden is another defining feature of EOCRC survivorship. A recent review of quality of life and psychosocial concerns in young-onset colorectal cancer emphasized that distress in this population is multidimensional, spanning physical symptoms, uncertainty about the future, disrupted identity, parenting concerns, career interruption, altered intimate relationships, and persistent fear of recurrence [47]. Earlier scoping work also found that younger patients often report greater psychosocial disruption than older patients, particularly in the domains of emotional distress, social functioning, and the challenge of living with cancer during a life stage usually associated with family formation and employment stability [50]. These burdens are compounded by the fact that many EOCRC patients look “too young to be ill,” which may reduce validation from employers, peers, and even healthcare professionals [47, 50].

Financial toxicity is now recognized as a major survivorship issue in EOCRC rather than a peripheral socioeconomic concern [46, 47, 51]. A 2024 National Health Interview Survey analysis found that colorectal cancer is associated with substantial long-term financial toxicity and that this burden disproportionately affects patients diagnosed before the age of 50 years [51]. Likewise, findings from the ColoCare Study showed that younger colorectal cancer survivors were more likely to remain in the workforce during and after treatment, yet paradoxically reported greater financial hardship than older survivors [52]. This pattern likely reflects the intersection of ongoing employment obligations, treatment-related costs, loss of income, insurance instability, family financial responsibilities, and the long duration over which economic consequences may accumulate [51, 52]. In EOCRC, financial toxicity should therefore be understood as part of disease burden itself, not merely as an external consequence of treatment.

Health-related quality of life in EOCRC is shaped not only by psychosocial and economic strain, but also by treatment-specific functional sequelae [47]. Younger patients treated for rectal cancer may live for decades with altered bowel habits, low anterior resection syndrome, ostomy-related distress, urinary dysfunction, or sexual impairment [34, 47]. Qualitative research on EOCRC-related health-related quality of life has shown that survivors frequently describe a broad and prolonged impact on daily life, including difficulties with eating, social participation, intimacy, fatigue, body confidence, and the unpredictability of bowel symptoms [53]. These findings reinforce an important clinical point: survivorship in EOCRC is not merely about “living after cancer,” but often about adapting to persistent changes in bodily function and self-perception during early and middle adulthood [47, 53].

Taken together, current evidence suggests that survivorship in EOCRC requires a broader and more age-attuned model of care [34, 46–48, 51]. Follow-up should not be limited to recurrence surveillance and management of treatment toxicity in the narrow biomedical sense. Instead, high-quality survivorship care for younger adults with colorectal cancer should include timely fertility counselling, structured attention to sexual health, psychosocial support, assessment of financial distress, help with employment-related challenges, and long-term management of functional sequelae [34, 47–52]. In this setting, successful treatment cannot be defined solely by survival or disease-free intervals; it must also include preservation, restoration, or active support of the life domains most disrupted by cancer occurring too early [34, 47].

### Research Gaps and Future Directions

Despite the rapid expansion of the EOCRC literature, major uncertainties remain, and many of the most frequently repeated assumptions in the field are still supported more by plausibility than by definitive evidence [5, 8, 17]. This is particularly true for biologic interpretation. Although EOCRC is increasingly discussed as a distinct entity, current data do not support a single unified molecular phenotype, and the field still lacks a robust biologic classification that separates hereditary, sporadic, inflammation-associated, microbiome-associated, and metabolically driven EOCRC in a reproducible way [5, 8, 14, 17]. One of the most urgent research priorities is therefore to move beyond binary EOCRC-versus-later-onset comparisons and toward a more granular subclassification framework integrating age, tumour site, germline status, somatic alterations, transcriptomic features, epigenetics, and tumour microenvironment [5, 8, 14, 17].

A second major gap lies in exposure science. Epidemiologic data strongly support a birth-cohort effect, and emerging models suggest that EOCRC may be shaped by early-life metabolic, dietary, microbial, and environmental influences [4, 5, 8]. However, direct prospective evidence linking specific prediagnostic exposures across the life course with downstream EOCRC risk remains limited [5, 8, 2, 3]. Future studies should therefore prioritize longitudinal cohorts capable of integrating childhood and adolescent exposures, antibiotic history, obesity trajectories, dietary patterns, microbiome development, inflammatory states, and environmental toxicants [4, 5, 8]. Without this type of design, the field risks continuing to generate compelling but only partially testable narratives about causation.

The microbiome is one of the most promising areas in EOCRC research, but it remains a field in transition from association to mechanism [5, 6]. Recent genomic evidence linking colibactin-associated mutational signatures to earlier age at diagnosis represents an important advance, yet many questions remain unanswered [6]. It is still unclear which patients are most affected by microbiome-associated mutagenesis, when the critical exposure window occurs, whether these findings generalize across populations, and whether microbiome-related signatures define a clinically meaningful EOCRC subtype [5, 6]. Future work should combine metagenomics, mutational signature analysis, host genomics, metabolomics, and prospective biospecimen collection to clarify whether microbiome-informed prevention or interception strategies are realistically achievable [5, 6].

Another major research priority is improvement of early detection in symptomatic younger adults. A growing literature shows that EOCRC diagnosis is often delayed despite the presence of red-flag symptoms, yet evidence-based diagnostic pathways for adults younger than screening age remain underdeveloped [4, 10]. The role of FIT in symptomatic younger populations is promising, but still requires broader validation across healthcare systems, symptom profiles, and prevalence settings [10]. Prospective implementation studies are needed to determine how best to combine symptoms, FIT, family history, laboratory abnormalities, and perhaps EHR-based prediction tools to reduce missed opportunities without overwhelming endoscopy services [4, 10].

Risk-stratified screening is another attractive but incompletely developed frontier. Current age-based screening remains necessary, but it is too blunt to address the full EOCRC problem [4, 39]. Future research should evaluate whether combinations of clinical risk factors, polygenic risk, family history, metabolic markers, stool-based biomarkers, and digital health data can identify younger adults at sufficiently elevated risk to justify earlier intervention [4, 39]. The challenge will not only be predictive performance, but also implementation, equity, cost-effectiveness, and avoidance of new disparities. A model that works well statistically but is inaccessible in real-world practice will not solve the EOCRC burden [39].

Survivorship research in EOCRC also remains underdeveloped relative to the scale of the problem. Fertility, sexual health, body image, employment disruption, financial toxicity, and long-term functional sequelae are increasingly recognized as core outcomes in younger patients, yet they are still not consistently embedded into prospective colorectal cancer research [34, 51]. Future trials and cohort studies should treat these domains not as optional quality-of-life appendices, but as formal endpoints relevant to treatment choice and long-term value of care [34, 51]. This is especially important in rectal cancer, where function-preserving strategies may confer major lifelong benefit even when traditional oncologic outcomes appear similar [34].

Finally, the EOCRC field needs greater methodological standardization. Studies continue to vary in age cutoffs, definitions of family history, inclusion of hereditary syndromes, treatment eras, and separation of colon versus rectal cancer, making cross-study comparison difficult [5, 8, 17]. Harmonized definitions, internationally comparable datasets, and prospective multi-institutional consortia will be essential if EOCRC research is to move from descriptive observation to clinically actionable knowledge [8, 17]. The next phase of the field should not simply produce more EOCRC studies, but better-structured EOCRC studies.

## Conclusion

EOCRC is no longer a marginal or purely epidemiologic curiosity. It has emerged as a clinically consequential and biologically complex form of colorectal cancer that challenges conventional assumptions about age, carcinogenesis, screening, and survivorship [4, 12, 13]. The available evidence suggests that EOCRC is not simply later-onset CRC occurring earlier, but a heterogeneous group of diseases shaped by the interplay of hereditary susceptibility, metabolic dysfunction, diet and lifestyle, microbiome-associated mutagenesis, inflammatory factors, and diagnostic delay [4, 12, 13, 17].

Current prevention and detection models remain only partially adapted to this reality. Lowering the screening age to 45 years was an important step, but it does not address the substantial proportion of EOCRC that arises before routine screening eligibility [3]. For many younger adults, diagnosis still depends on recognition of symptoms, timely referral, hereditary risk assessment, and increasingly, more nuanced risk-adapted strategies [3, 4]. At the same time, treatment of EOCRC must be understood not only in terms of oncologic control, but also in relation to fertility, sexual health, long-term function, financial toxicity, and the broader disruption caused by cancer during early and middle adulthood [13, 34].

The central challenge ahead is therefore not merely to describe EOCRC more thoroughly, but to rethink colorectal cancer care across the life course. Future progress will depend on more precise biologic subclassification, stronger exposure-linked epidemiology, earlier and more reliable symptomatic detection, and survivorship models that reflect the realities of younger patients [3, 4, 12, 13, 17, 34]. In this sense, EOCRC should be viewed not only as a growing clinical problem, but also as a signal that current paradigms of colorectal cancer prevention and care require substantial refinement.

## Data Availability

No datasets were generated or analysed during the current study.

## Key References

- Sung H, Siegel RL, Laversanne M, Jiang C, Morgan E, Zahwe M, Cao Y, Bray F, Jemal A: Colorectal cancer incidence trends in younger versus older adults: an analysis of population-based cancer registry data. The Lancet Oncology 2025, 26(1):51-63.
  - ○ This population-based analysis across 50 countries and territories provides one of the strongest contemporary epidemiologic foundations for EOCRC research. It demonstrates that the rise in colorectal cancer among younger adults is a global phenomenon and not merely a local artefact of screening or detection.
- Díaz-Gay M, Dos Santos W, Moody S, Kazachkova M, Abbasi A, Steele CD, Vangara R, Senkin S, Wang J, Fitzgerald S: Geographic and age variations in mutational processes in colorectal cancer. Nature 2025, 643(8070):230-240.
  - ○ This landmark genomic study links colibactin-associated mutational signatures with younger-onset colorectal cancer and provides mechanistic support for microbiome-mediated early carcinogenesis. It is particularly important because it connects early-life microbial genotoxic exposure with early driver events in colorectal tumorigenesis.
- Du M, Drew DA, Goncalves MD, Cao Y, Chan AT: Early-onset colorectal cancer as an emerging disease of metabolic dysregulation. Nature Reviews Endocrinology 2025, 21(11):686-702.
  - ○ This review offers a major conceptual framework for understanding EOCRC as a life-course disease linked to obesity, insulin resistance, metabolic dysfunction, and related host–microbiome interactions. It is highly relevant to the interpretation of birth-cohort effects and changing generational exposures.
- Fritz CD, George M, Carethers JM, Cao Y: Approach toward early detection and prevention of early-onset colorectal cancer. Annual Review of Medicine 2025, 77.
  - ○ This article provides a clinically oriented synthesis of prevention and early-detection strategies for EOCRC. It is important because it highlights the limitations of age-based screening alone and supports a broader approach incorporating family history, symptoms, risk stratification, and precision prevention.
- Demb J, Kolb JM, Dounel J, Fritz CD, Advani SM, Cao Y, Coppernoll-Blach P, Dwyer AJ, Perea J, Heskett KM: Red flag signs and symptoms for patients with early-onset colorectal cancer: a systematic review and meta-analysis. JAMA Network Open 2024, 7(5).
  - ○ This systematic review and meta-analysis defines the symptom profile most strongly associated with EOCRC, including haematochezia, abdominal pain, altered bowel habits, and anaemia. It is clinically important because it reframes EOCRC as a frequently symptomatic disease in which diagnostic delay often reflects under-recognition rather than absence of warning signs.
- Cornish AJ, Gruber AJ, Kinnersley B, Chubb D, Frangou A, Caravagna G, Noyvert B, Lakatos E, Wood HM, Thorn S: The genomic landscape of 2,023 colorectal cancers. Nature 2024, 633(8028):127-136.
  - ○ This large genomic analysis provides important context for interpreting age- and site-associated molecular features in colorectal cancer. It supports the view that EOCRC should not be treated as a single molecular entity, but rather as a heterogeneous group of tumours with overlapping and distinct biological features.
- Benson AB, Venook AP, Adam M, Chang G, Chen YJ, Ciombor KK, Cohen SA, Cooper HS, Deming D, Garrido-Laguna I et al: NCCN Guidelines® Insights: Rectal Cancer, Version 3.2024. Journal of the National Comprehensive Cancer Network 2024, 22(6):366-375.
  - ○ This guideline update is important for EOCRC because younger patients are disproportionately affected by distal colon and rectal cancers. It provides clinically relevant context for total neoadjuvant therapy, response-adapted care, and organ-preserving strategies such as watch-and-wait in selected patients.
- Jiang Q, Xu X, Sun P, Hua H: Sexual and Reproductive Health of Patients With Early-Onset Colorectal Cancer. Clinical and Translational Gastroenterology 2025, 16(10).
  - ○ This review addresses a key survivorship domain that is often underrepresented in EOCRC management. It is important because fertility, sexuality, pelvic function, and reproductive planning are central to age-attuned care in younger colorectal cancer survivors.
